# Integration of network toxicology and transcriptomics reveals the potential regulatory role of the 'Arecoline/4-NQO-AKT1-m6A factor' axis in the development of OSCC

**DOI:** 10.3389/fonc.2026.1739418

**Published:** 2026-03-23

**Authors:** Yongle Qiu, Yamei Wang, Jiahong Zhao, Yi Cheng, Yang Liu, Hui Xu, Zhizheng Zhuang, Feifei Lv

**Affiliations:** 1 Department of Stomatology, The Fourth Hospital of Hebei Medical University, Shijiazhuang, Hebei, China; 2 Department of Stomatology, Qinhuangdao Haigang Hospital, Qinhuangdao, Hebei, China; 3 Department of Dermotology, The Fourth Hospital of Hebei Medical University, Shijiazhuang, Hebei, China; 4 Department of Stomatology, The Second Hospital of Shijiazhuang, Shijiazhuang, Hebei, China; 5 Department of Stomatology, Affiliated Hospital of Hebei University, Baoding, Hebei, China

**Keywords:** 4-NQO, AKT1, arecoline, M6A, oral squamous cell carcinoma (OSCC)

## Abstract

**Objective:**

The synergistic carcinogenic effect of areca nut chewing and tobacco exposure is a major risk factor for oral squamous cell carcinoma (OSCC). However, the molecular mechanisms, particularly related to N6-methyladenosine (m6A) modification, remain unclear. This study explores the potential mechanisms by which arecoline and 4-nitroquinoline-1-oxide (4-NQO) induce OSCC, focusing on m6A regulatory factors and key targets.

**Methods:**

Transcriptional data were obtained from Gene Expression Omnibus (GEO), with differentially expressed genes (DEGs) identified using the limma package. The intersection of DEGs and 21 m6A regulators was analyzed, and their prognostic relevance was validated with The Cancer Genome Atlas (TCGA) data. Arecoline and 4-NQO targets were identified through online databases, and compound-target and protein-protein interaction (PPI) networks were constructed. Core targets were selected using Degree, MCC, and FRIENDS algorithms. Spearman analysis assessed correlations with m6A factors, and molecular docking was performed to verify binding properties.

**Results:**

Heterogeneous Nuclear Ribonucleoprotein C (HNRNPC), AlkB Homolog 5 (ALKBH5), and ELAV Like RNA Binding Protein 1 (ELAVL1) were identified as key genes. High HNRNPC expression correlated with poor prognosis. AKT Serine/Threonine Kinase 1 (AKT1) was the core target across algorithms, with significant correlations between AKT1 and m6A factors. Molecular docking indicated potential binding between AKT1 and the compounds.

**Conclusion:**

This study establishes a regulatory network linking arecoline/4-NQO, AKT1, m6A factors, and OSCC, and identifies key molecular targets and mechanisms underlying the carcinogenic process. These findings provide a theoretical foundation for understanding the pathogenesis of OSCC and developing targeted strategies for early intervention and treatment.

## 1 Introduction

Oral squamous cell carcinoma (OSCC) is a highly prevalent and lethal subtype of head and neck malignancies, with its development closely linked to prolonged exposure to carcinogenic substances, posing a significant threat to both oral health and quality of life (1, 2). Among the primary risk factors for OSCC, the consumption of areca catechu and tobacco use are key contributors, and notably, co-exposure to both is common in the population. However, most current research focuses on the carcinogenic mechanisms of individual carcinogens, with limited insight into the molecular pathways underlying their synergistic carcinogenic effects, especially in the context of post-transcriptional regulation.

The carcinogenic activity of betel nut primarily arises from its alkaloids, with arecoline comprising 80-90% of the total alkaloid content and acting as the principal carcinogenic component (3). The International Agency for Research on Cancer (IARC) has classified arecoline as a Group 2B carcinogen (4), demonstrating that it promotes carcinogenesis through mechanisms such as inducing reactive oxygen species (ROS) accumulation in oral mucosa, activating the NF-κB inflammatory pathway, inhibiting apoptosis, and causing DNA damage, which in turn facilitates the malignant transformation of oral submucous fibrosis (OSF) into OSCC (5). 4-Nitroquinoline 1-oxide (4-NQO), a classical chemical carcinogen, mimics the carcinogenic components of tobacco smoke, such as nitrosamines and polycyclic aromatic hydrocarbons, by forming stable adducts with genomic DNA that lead to DNA damage and gene mutations, ultimately inducing OSCC (6, 7). The tumor model established by 4-NQO closely resembles tobacco-related OSCC in terms of both pathological morphology and gene expression, making it a widely used tool for OSCC mechanistic research.

N6-methyladenosine (m6A) modification is the most prevalent post-transcriptional modification of mRNA in eukaryotes. It regulates target mRNA stability, translation efficiency, and splicing through a network of "writers," "erasers," and "readers," thereby influencing key carcinogenic processes such as cell proliferation, inflammation, immune regulation, and DNA damage repair (8, 9). Previous studies have established that dysregulation of m6A modification is closely associated with the progression of OSCC. For instance, METTL3 promotes OSCC by modulating the miR-146a-5p/SMAD4 axis (10), and FTO sensitizes OSCC to ferroptosis by inhibiting ACSL3 and GPX4 (11). However, no studies have yet clarified whether co-exposure to 4-NQO and arecoline disrupts the m6A modification system to regulate the expression of key carcinogenic genes, thereby exacerbating OSCC progression. This "post-transcriptional regulatory gap" significantly limits our understanding of the synergistic carcinogenic mechanisms of these two agents.

Notably, most existing studies focus on single carcinogen exposure, while oral carcinogenesis in clinical practice is often driven by multiple risk factors acting in concert. Areca nut use and tobacco exposure frequently coexist in high-risk populations, and their synergistic effect significantly increases the risk of OSCC compared with single exposure. Therefore, using a combined model of arecoline and 4-NQO more closely mimics the real-world etiology of OSCC and has greater translational relevance. From a mechanistic perspective, arecoline and 4-NQO exert carcinogenic effects through distinct pathways: arecoline mainly induces oxidative stress, inflammation, and DNA damage, whereas 4-NQO acts as a classic DNA alkylating agent that mimics tobacco-related carcinogenesis. Their combination allows for a more comprehensive simulation of the complex molecular network underlying multi-factorial carcinogenesis, rather than focusing on pathway-specific changes induced by a single agent. For m6A-related mechanisms, this combined exposure strategy helps identify robust and clinically relevant m6A regulatory axes that are consistently dysregulated under multi-factor carcinogenic stress, rather than context-specific or random changes under single-agent exposure. This design therefore enhances the reliability and generalizability of the identified core regulatory targets.

Network toxicology, an emerging technology integrating bioinformatics and toxicology, can uncover associations between "toxicants, targets, and diseases" from public databases, constructing molecular interaction networks that efficiently identify key targets and enriched pathways. This approach provides valuable insights into the mechanisms of complex carcinogen co-exposure (12–14). Therefore, as an original bioinformatics research, this study integrates transcriptomic data (GSE64271, GSE74530, GSE38206), network toxicology, protein-protein interaction (PPI) network analysis, and molecular docking to systematically investigate the relationships between m6A core regulatory factors, key targets, and their roles in the carcinogenic process induced by 4-NQO and arecoline. The aim is to uncover the potential regulatory chain of "carcinogen - core target - m6A dysregulation - OSCC," providing a theoretical foundation for early intervention and target development in OSCC.

## 2 Materials and methods

### 2.1 Transcriptomic data acquisition and differential expression analysis

Three transcriptomic datasets related to OSCC were downloaded from the Gene Expression Omnibus (GEO, https://www.ncbi.nlm.nih.gov/geo/).

The GSE64271 dataset includes 5 normal tongue tissue samples and 7 OSCC tumor tissue samples induced by 4-NQO combined with arecoline in C57BL/6 mice. The detection platform for this dataset is Illumina Mouse Ref-8 v2.0 Expression BeadChip (GPL6885). This dataset was processed using the GEO2R online tool, which employs a standard limma-based analysis pipeline, including background correction, quantile normalization, and batch effect correction to ensure data comparability.

GSE74530 and GSE38206 were used to construct an OSCC disease-related target library. Data preprocessing for GSE74530 and GSE38206 was performed using R version 4.2.1 with the affy and limma packages, using the identical preprocessing pipeline as described above. This included background correction by the RMA algorithm, quantile normalization, and probe annotation. Probes were mapped to gene symbols, with the average expression value taken for redundant probes corresponding to the same gene.

All datasets were analyzed using a “tumor vs. normal tissue” comparison model, and differentially expressed genes (DEGs) were selected with adjusted P values (adj. P. Val) < 0.05 and |log_2_FC| > 1. Volcano plots were generated using the ggplot2 package to validate the reliability of the differential analysis.

### 2.2 Intersection analysis of m6A core regulators and differential genes

To identify m6A-related DEGs, a literature search of PubMed was conducted using the keywords "m6A core regulator" and "Writer/Eraser/Reader family." A total of 21 validated m6A modification core regulators were collected and classified into the Writer, Eraser, and Reader families based on their functions. A Venn diagram was used to intersect the DEGs from GSE64271 with these 21 m6A core regulators to obtain the "m6A-related DEGs." Additionally, boxplots were generated using Xiantao Academic Online Tools to compare gene expression between tumor and normal tissues. Additionally, all survival analyses, including univariate Kaplan–Meier analysis and Cox regression, were performed using Xiantao Academic Online Tools.

### 2.3 Network toxicology screening of potential compound targets

Arecoline and 4-NQO were selected as study compounds. Potential human (Homo sapiens) targets were screened using eight network toxicology databases: SEA, SwissTargetPrediction, STITCH, TargetNET, CTD, ChemBL, PharMapper, and BATMAN. Duplicates were removed, and the final candidate target set for each compound was compiled. The dplyr package in R was used to integrate and standardize gene symbols. Venn diagrams were generated to identify common and unique targets between arecoline and 4-NQO, providing a basis for investigating their synergistic effects. A "Compound-Target" interaction network was constructed using Cytoscape 3.10.2.

### 2.4 Gene enrichment analysis

Multidimensional enrichment analyses were conducted on the union of compound targets and the common "compound-OSCC" targets using Xiantao Academic Online Tools. Gene symbols were converted to ENTREZ IDs to ensure analysis accuracy. Gene Ontology (GO) functional enrichment (covering biological processes (BP), cellular components (CC), molecular functions (MF)) and Kyoto Encyclopedia of Genes and Genomes (KEGG) pathway enrichment (via https://www.genome.jp/kegg/) were performed with the same parameters: significance threshold of adjusted P value (padj) < 0.05, and Benjamini–Hochberg (BH) method for multiple testing correction (to control false positive rates).Hallmark gene set enrichment (using MSigDB’s 50 hallmark sets) also adopted the same BH correction method and padj < 0.05 threshold; results were visualized via chord diagrams to show target-hallmark term associations.

### 2.5 Construction and intersection of the OSCC disease-related target library

The GeneCards database (https://www.genecards.org/) was queried with the keyword "Oral Squamous Cell Carcinoma" to retrieve disease-related genes. Additionally, DEGs from the GSE74530 and GSE38206 datasets were extracted. The union of these three datasets was used to construct an OSCC disease-related target library. A Venn diagram was generated to find the intersection between "shared targets of arecoline and 4-NQO" and the OSCC disease-related target library. The resulting common targets were imported into the STRING database (https://string-db.org/), with parameters set for Homo sapiens and a confidence score > 0.9. Isolated targets without interactions were excluded. The Protein-Protein Interaction (PPI) network was visualized in Cytoscape 3.10.2, and gene enrichment analysis was repeated to clarify the functional roles and pathway associations of these targets in OSCC.

### 2.6 Core target selection in the PPI network

The FRIENDS algorithm, implemented via Xiantao Academic Online Tools (https://www.xiantao.love/), was used to filter core targets based on gene co-expression correlation (15–17). The FRIENDS algorithm is based on the following principle: by ranking gene similarity scores and identifying structural breaks in gene interaction profiles, a gene that interacts extensively with multiple other genes in a pathway or functional module (e.g., through protein-protein interactions, co-expression, or regulation) can be identified as a hub gene, which plays a crucial regulatory role in the pathway’s function. We ranked the genes based on their similarity scores. In this study, the FRIENDS algorithm was employed to identify key molecular interactions associated with arecoline/4NQO exposure and abnormalities in OSCC. Core targets in the PPI network of common targets were further selected using the “Degree” and “MCC” algorithms from the CytoHubba plugin in Cytoscape. The top 5 ranked genes from each algorithm were identified as important targets, and the intersection of results from the three algorithms was used to determine the core regulatory targets for OSCC.

### 2.7 Correlation analysis of core targets with m6A differential genes

Based on transcriptomic data from the TCGA database for OSCC, we analyzed the correlation between the expression of key targets and m6A-related DEGs (HNRNPC, ALKBH5, ELAVL1). Prior to correlation analysis, we assessed the normality of the expression data for these key genes using the Shapiro-Wilk test. Based on the results of the normality test, we ultimately chose the non-parametric Spearman rank correlation analysis to ensure the robustness of the statistical analysis. The correlation coefficients (r) and corresponding p-values were calculated to determine the strength and statistical significance of the expression associations among the genes.

### 2.8 Molecular docking validation

Molecular docking analysis was conducted using compounds obtained from the PubChem database. For compounds lacking 3D structures, structure generation was performed with Chem3D software, followed by conversion to PDB format using OpenBabel. AutoDockTools was used to preprocess the compounds, which included adding polar hydrogens, assigning Gasteiger charges, and defining rotatable bonds before saving the files in PDBQT format. The crystal structures of core target proteins were retrieved from the Protein Data Bank (PDB, https://www.rcsb.org/), processed by removing water molecules and ligands, adding polar hydrogens, and assigning Kollman charges, and saved as PDBQT files. The grid box for molecular docking was centered on the original ligand-binding pocket of each target protein, with the grid center coordinates determined based on the crystal structure of the corresponding protein; the grid size was set to 40 × 40 × 40 Å and the grid spacing was 0.375 Å, covering the complete active pocket of the protein. The docking exhaustiveness was set to 20 to ensure sufficient sampling of the ligand binding conformations, and all other docking parameters were adopted as the default settings of the software. Binding modes and energies were calculated using AutoDock Vina, and the results were visualized and analyzed with PyMOL to assess binding mechanisms and key interactions.

## 3 Results

### 3.1 Intersection of differential genes from GSE64271 and m6A core regulatory factors

**Figure 1:**
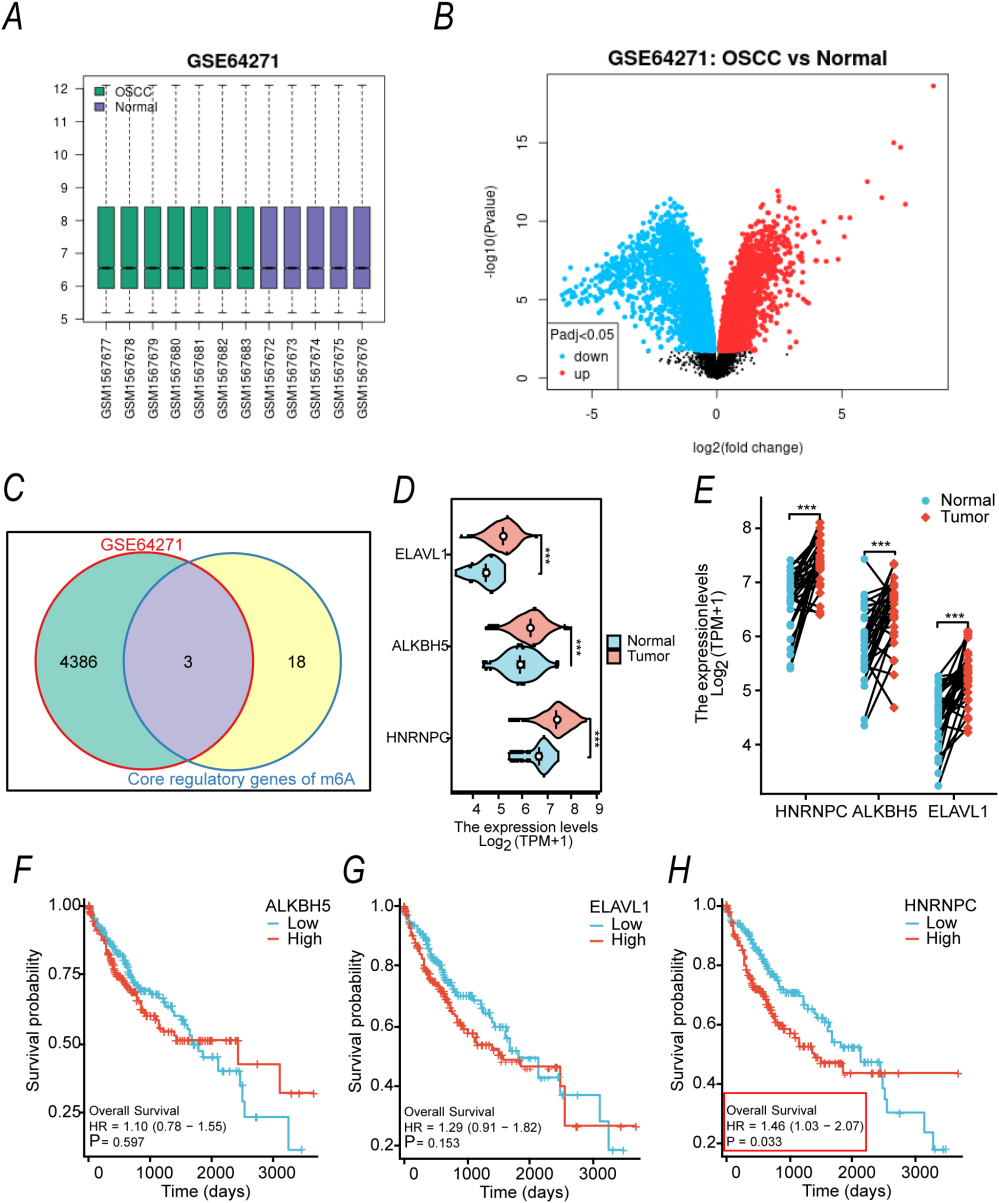
Identification of m6A Core Regulatory Factors Associated with OSCC Induced by Arecoline Combined with 4-NQO **(A)** Sample distribution in the GSE64271 dataset. **(B)** Volcano plot of differentially expressed genes (DEGs) in the GSE64271 dataset (adjusted P < 0.05, |log_2_FC| > 1). Red dots represent upregulated genes, blue dots represent downregulated genes, and gray dots represent genes with no significant differential expression. **(C)** Venn diagram showing the intersection of DEGs from GSE64271 with 21 known m6A core regulatory factors. **(D, E)** Boxplots comparing the expression levels of target genes (HNRNPC, ALKBH5, ELAVL1) between OSCC tumor and normal tissues based on TCGA database. **(D)** Non-paired sample plot; **(E)** Paired sample plot. **(F-H)** Kaplan-Meier survival curves based on TCGA database showing the association of HNRNPC **(F)**, ALKBH5 **(G)**, and ELAVL1 **(H)** expression with overall survival in OSCC patients.

To identify potential m6A regulatory factors involved in the carcinogenesis of OSCC induced by arecoline and 4-NQO, we first performed differential gene expression analysis on the GSE64271 dataset, which includes OSCC tumor tissues and normal tongue tissues from mice exposed to both 4-NQO and arecoline. The sample distribution (**Figure 1A**) and volcano plot (**Figure 1B**) revealed significant differential expression between tumor and normal tissues. The intersection between the differential genes and 21 known m6A core regulatory factors (encompassing the "Writer," "Eraser," and "Reader" families) was analyzed. The Venn diagram (**Figure 1C**) showed that three genes, HNRNPC, ALKBH5, and ELAVL1, were common between the DEGs and the m6A core regulatory factors.

To validate these findings, we further analyzed gene expression differences between OSCC and normal tissues in the TCGA database. Box plots (**Figures 1D, E**) indicated that the expression of HNRNPC, ALKBH5, and ELAVL1 was significantly higher in tumor tissues compared to normal tissues (***p< 0.001). Survival analysis (**Figures 1F–H**) revealed that high expression of HNRNPC was associated with significantly reduced overall survival in OSCC patients (hazard ratio [HR] = 1.46, 95% confidence interval [CI] 1.03–2.07, P = 0.033), while the expression levels of ALKBH5 and ELAVL1 were not significantly correlated with overall survival (P = 0.597, P = 0.153, respectively). The Cox regression results indicated that high expression of HNRNPC remained significantly associated with poor overall survival in OSCC patients, even after adjusting for clinical confounding factors, thus confirming its role as an independent prognostic predictor for OSCC (**Table 1**).

**Table 1:** Cox regression analysis of clinical pathological features and overall survival in OSCC patients.

Characteristics	Total (N)	Univariate analysis		Multivariate analysis	
		Hazard ratio (95% CI)	P value	Hazard ratio (95% CI)	P value
Pathologic T stage	304				
T1	29	Reference		Reference	
T2	99	1.756 (0.737 - 4.185)	0.204	1.848 (0.617 - 5.539)	0.273
T3	66	3.980 (1.677 - 9.447)	**0.002**	3.782 (1.268 - 11.280)	**0.017**
T4	110	3.717 (1.592 - 8.676)	**0.002**	3.568 (1.217 - 10.457)	**0.020**
Pathologic N stage	275				
N0	117	Reference		Reference	
N1	50	0.747 (0.400 - 1.394)	0.359	0.758 (0.405 - 1.421)	0.388
N2	106	2.222 (1.499 - 3.294)	**< 0.001**	1.911 (1.258 - 2.904)	**0.002**
N3	2	3.902 (0.941 - 16.184)	0.061	2.438 (0.577 - 10.296)	0.225
Age	329				
<= 60	156	Reference		Reference	
> 60	173	1.330 (0.961 - 1.840)	0.085	1.521 (1.044 - 2.215)	**0.029**

The bold P values represent statistically significant results (P < 0.05), indicating that these clinicopathological features are significantly associated with overall survival in OSCC patients.

### 3.2 Potential targets of arecoline and 4-NQO, and network construction

**Figure 2:**
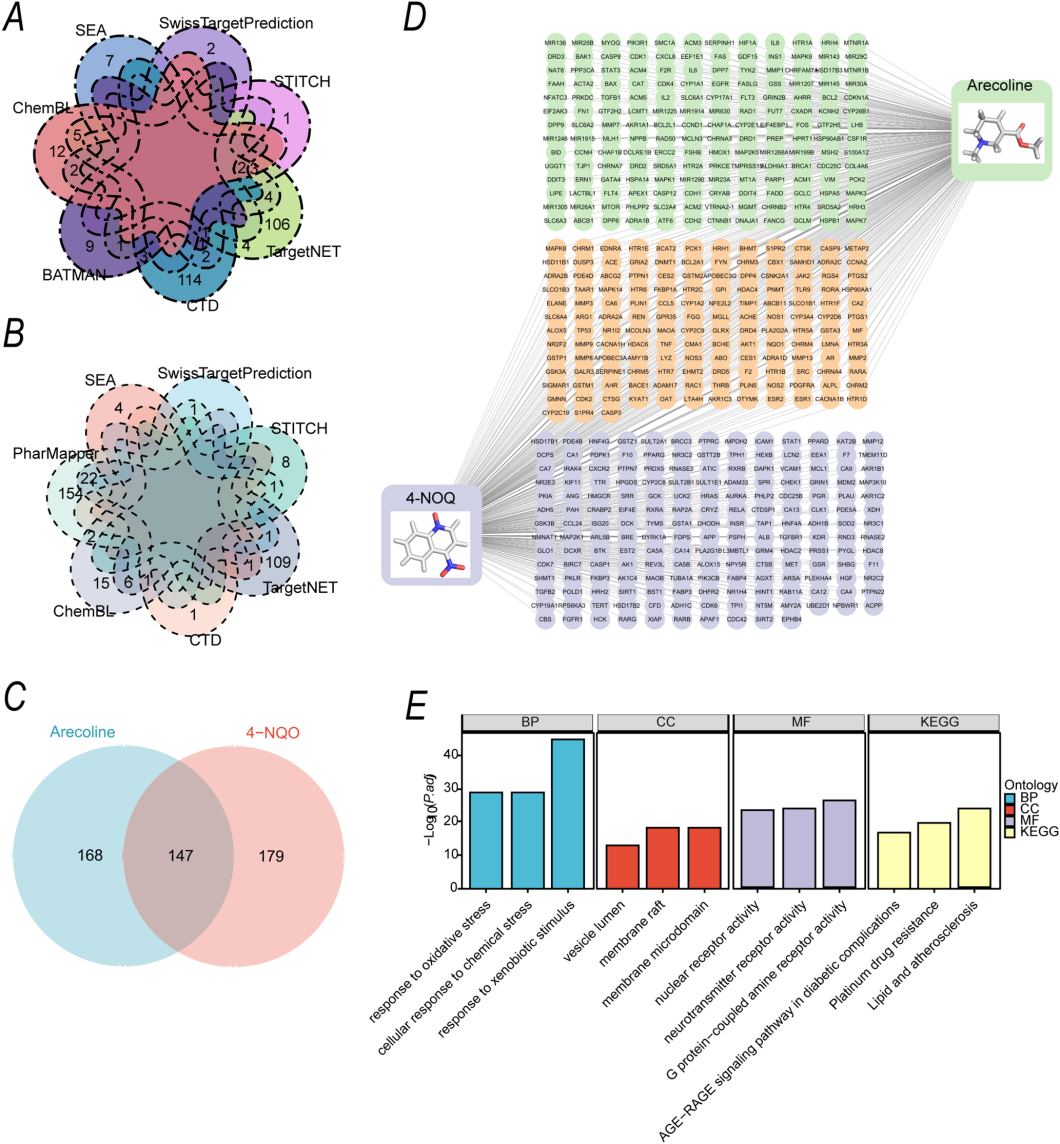
Potential Targets of Arecoline and 4-NQO and Construction of the "Compound-Target" Interaction Network **(A)** Distribution of potential targets for arecoline, identified by integrating eight databases: SEA, SwissTargetPrediction, STITCH, TargetNET, CTD, ChemBL, PharMapper, and BATMAN. **(B)** Distribution of potential targets for 4-NQO, identified using the same multi-database integration strategy. **(C)** Venn diagram showing the intersection of potential targets for arecoline and 4-NQO. Targets on the left are unique to arecoline, those on the right are unique to 4-NQO, and the overlap in the center represents shared targets. **(D)** "Compound-Target" interaction network constructed using Cytoscape 3.10.2. Rectangular nodes represent compounds, circular nodes represent targets, and edges indicate interactions between compounds and targets. **(E)** GO functional enrichment analysis and KEGG pathway enrichment analysis of the union of arecoline and 4-NQO targets. GO enrichment includes biological processes (BP), cellular components (CC), and molecular functions (MF) (adjusted P < 0.05).

To identify the potential targets of arecoline and 4-NQO, we queried several databases, including SEA (18), SwissTargetPrediction (19–21), STITCH, TargetNET (22), CTD (23), ChemBL (24, 25), PharMapper (26) and BATMAN (27) visualized the results using a Venn diagram. A total of 315 potential targets for arecoline (**Figure 2A**) and 326 potential targets for 4-NQO (**Figure 2B**) were identified. The Venn diagram (**Figure 2C**) showed that arecoline had 168 unique targets, 4-NQO had 179 unique targets, and 147 common targets were shared between the two. This suggests that arecoline and 4-NQO may exert a synergistic carcinogenic effect through overlapping targets. Based on these targets, a "compound-target" interaction network was constructed using Cytoscape 3.10.2 (**Figure 2D**), which visually depicted the relationships between arecoline, 4-NQO, and their respective targets. Additionally, GO and KEGG pathway enrichment analyses were conducted for the union of both sets of targets. Enrichment results (**Figure 2E**) showed that the biological processes of the common targets were significantly enriched in "oxidative stress response" and "cellular response to chemical stress," while cellular components were associated with "vesicle lumen" and "membrane raft," and molecular functions included "nuclear receptor activity" and "neurotransmitter receptor activity." KEGG pathway analysis revealed significant associations with pathways related to "AGE-RAGE signaling in diabetic complications," "lipid metabolism and atherosclerosis," and other cancer- and stress-related pathways.

### 3.3 Construction of OSCC disease-related target library

**Figure 3:**
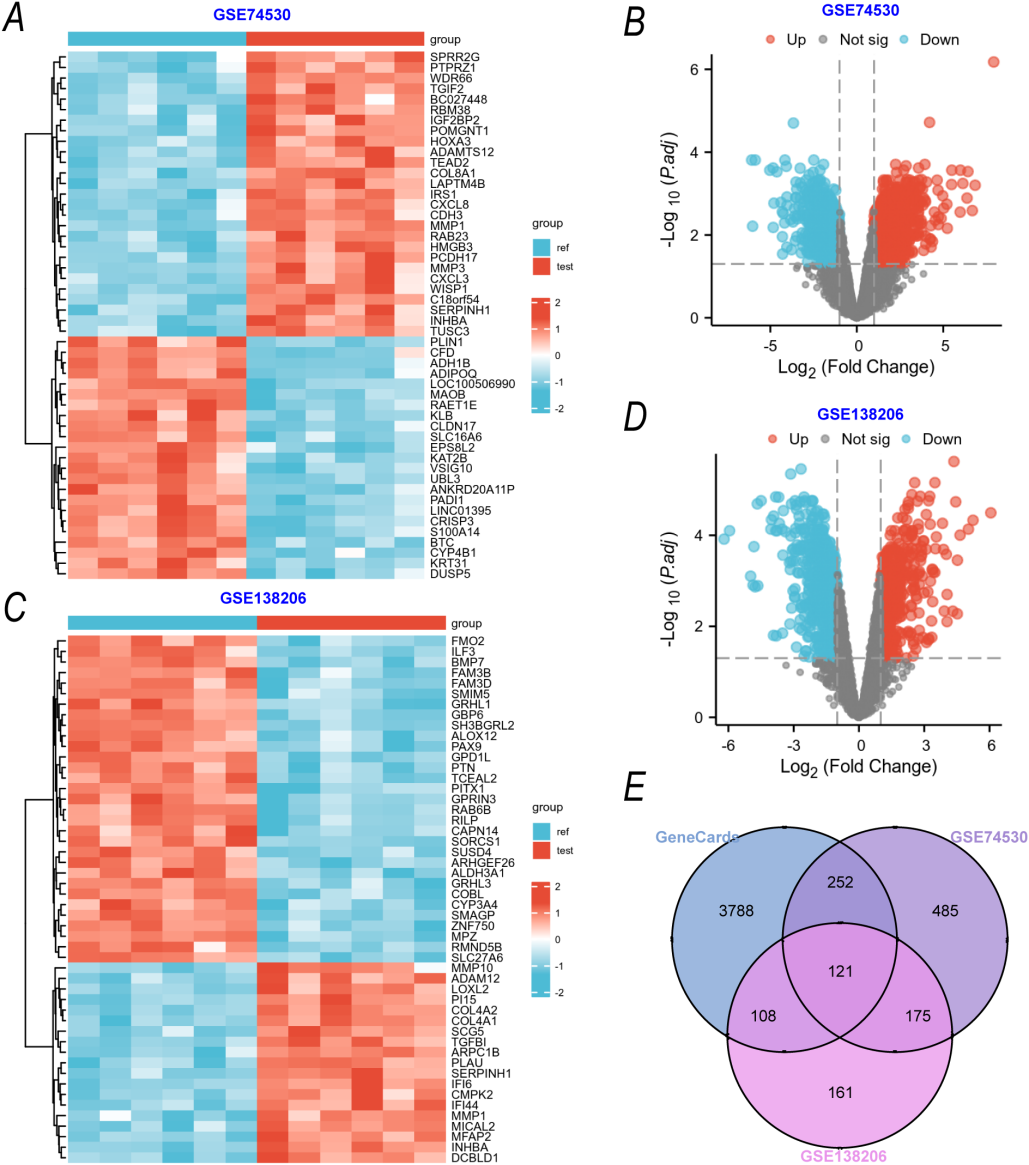
Construction of an OSCC Disease-Related Target Library **(A, C)** Gene expression heatmaps for the GSE74530 **(A)** and GSE38206 **(C)** datasets. Rows represent genes, columns represent samples (red for OSCC tumor tissues, blue for normal tissues), and the color intensity reflects gene expression levels (log_2_-transformed). **(B, D)** Volcano plots of DEGs from the GSE74530 **(B)** and GSE38206 **(D)** datasets (adjusted P < 0.05, |log_2_FC| > 1). **(E)** Venn diagram showing the intersection of OSCC-related genes annotated by the GeneCards database, DEGs from GSE74530, and DEGs from GSE38206.

To identify OSCC disease-related targets, we performed an analysis using the GeneCards database in conjunction with the GSE74530 and GSE38206 human OSCC transcriptomic datasets. The gene expression heatmaps (**Figures 3A, C**) revealed significant differential gene expression between OSCC tumor tissues and normal tissues in both datasets. The volcano plots (**Figures 3B, D**) further quantified the gene expression changes, showing 389 upregulated genes and 644 downregulated genes in GSE74530, and 235 upregulated genes and 330 downregulated genes in GSE38206. A Venn diagram (**Figure 3E**) was used to analyze the intersection of "GeneCards OSCC-related genes," "GSE74530 differential genes," and "GSE38206 differential genes." The results indicated that there were 5090 overlapping genes, which were subsequently considered as potential targets for OSCC.

### 3.4 Potential targets of arecoline and 4-NQO in OSCC

**Figure 4:**
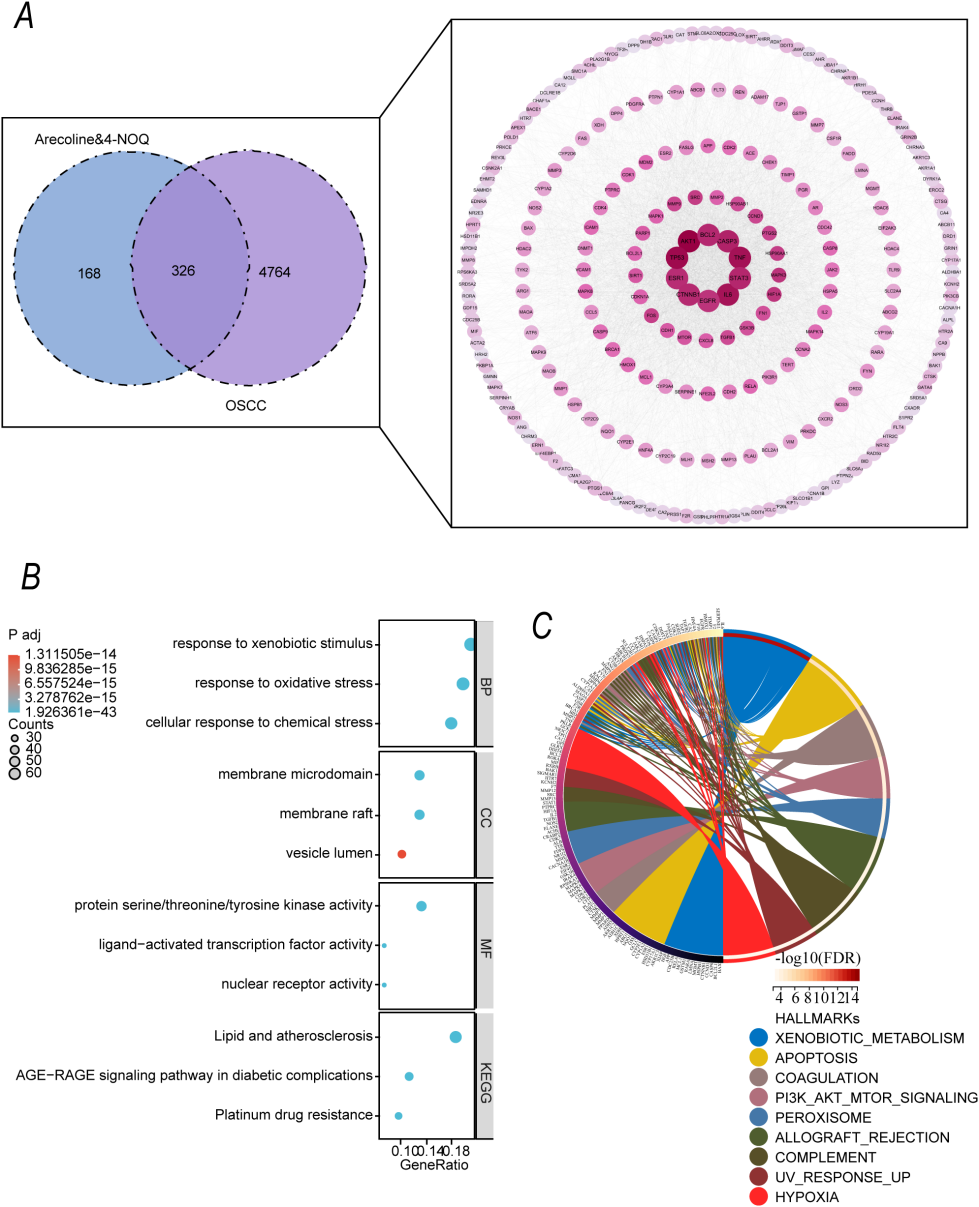
Potential Targets Regulated by Arecoline and 4-NQO in OSCC **(A)** Venn diagram showing the intersection of "shared targets of arecoline and 4-NQO" and "OSCC disease-related targets." The intersecting targets are considered key mediators of OSCC regulation by both compounds. **(B)** GO functional enrichment analysis and KEGG pathway enrichment analysis of the intersecting targets (adjusted P < 0.05). **(C)** Hallmark gene set enrichment analysis chord diagram for the intersecting targets.

To explore the potential targets of arecoline and 4-NQO in OSCC, we intersected the targets of both compounds with the OSCC disease-related targets. The Venn diagram (**Figure 4A**) showed that 326 targets of arecoline and 4-NQO overlapped with OSCC disease-related targets, suggesting that these intersecting targets may be key mediators in the regulation of OSCC by both compounds. The subsequent PPI network illustrated the dense interactions between these intersecting targets, with high connectivity for core nodes.

Enrichment analysis of the targets revealed that the biological processes (BP) were significantly enriched in "response to exogenous stimulus," "oxidative stress response," and "cellular response to chemical stress" (padj < 0.05). The cellular components (CC) involved "membrane microdomain," "membrane raft," and "vesicle lumen," while molecular functions (MF) included "protein serine/threonine/tyrosine kinase activity" and "ligand-activated transcription factor activity" (padj < 0.05). KEGG pathway analysis showed significant associations with pathways such as "AGE-RAGE signaling in diabetic complications," "lipid metabolism and atherosclerosis," and "platinum drug resistance." (padj < 0.05) (**Figure 4B**). Additionally, enrichment analysis of the Hallmark gene sets (**Figure 4C**) revealed that these intersecting targets were involved in key tumor-related processes, including "heterologous substance metabolism," "apoptosis," and "PI3K-AKT-mTOR signaling." (padj < 0.05).

### 3.5 Core target selection from PPI network: identification of AKT1 as the hub gene

**Figure 5:**
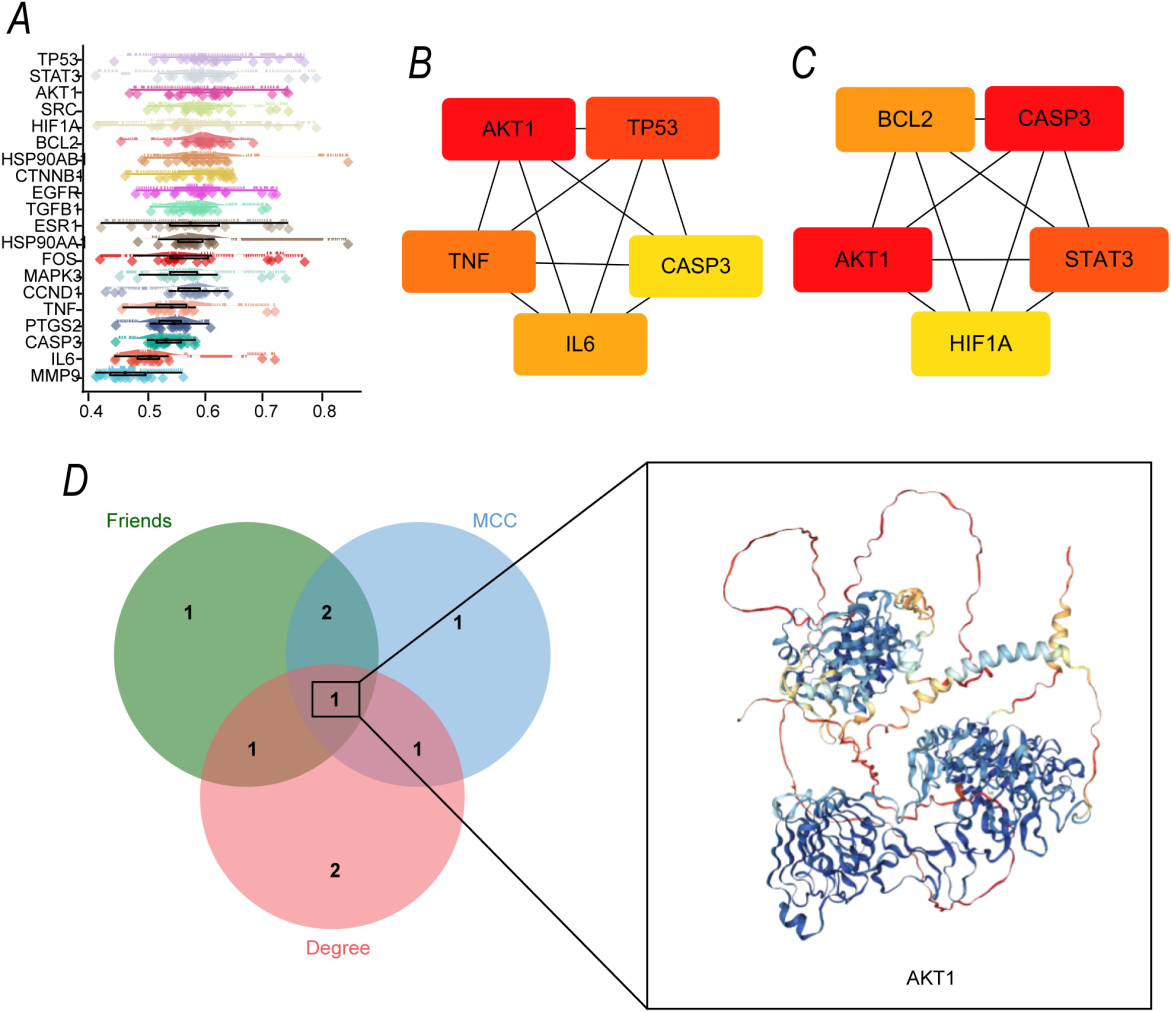
Identification of Core Targets in the PPI Network of Intersecting Targets **(A)** Connectivity ranking of the intersecting targets in the PPI network, generated using the FRIENDS algorithm, where connectivity reflects the regulatory associations of genes within the network. **(B)** Core targets in the intersecting targets' PPI network, identified using the "Degree" algorithm in the Cytoscape CytoHubba plugin. **(C)** Core targets in the intersecting targets' PPI network, identified using the "MCC" algorithm in the Cytoscape CytoHubba plugin. **(D)** Venn diagram showing the intersection of core target results obtained from the FRIENDS, Degree, and MCC algorithms.

To identify the core regulatory molecules involved in the interaction between the compounds and OSCC, we analyzed the PPI network of the intersecting targets using the FRIENDS algorithm. The degree-ranking diagram (**Figure 5A**) showed that genes such as TP53, STAT3, AKT1, and SRC had significantly higher connectivity than other nodes. Further analysis using the CytoHubba plugin in Cytoscape, employing two classic algorithms, identified the following core targets: the "Degree" algorithm (**Figure 5B**) highlighted AKT1, TP53, TNF, CASP3, and IL6 as potential core targets, while the "MCC" algorithm (**Figure 5C**) focused on BCL2, CASP3, AKT1, STAT3, and HIF1A.

To refine the core targets, a Venn diagram (**Figure 5D**) was constructed to analyze the intersection of the core targets identified by the FRIENDS, Degree, and MCC algorithms. The results showed that AKT1 was consistently identified as a core target across all three algorithms, and no other gene appeared in all three sets.

### 3.6 Correlation between AKT1 and m6A regulation genes

To investigate the association between AKT1 and the expression levels of DEGs involved in m6A regulation, we utilized transcriptomic data from the TCGA database for OSCC. First, we performed the Shapiro-Wilk test on the expression data of key genes (AKT1, HNRNPC, ALKBH5, ELAVL1) to determine the appropriate correlation analysis method. The results showed that the expression data for AKT1 (P = 5.1e-07) and HNRNPC (P = 0.0019) significantly deviated from normal distribution (P < 0.05). Therefore, we chose the non-parametric Spearman rank correlation analysis to ensure the robustness and consistency of the statistical inference, avoiding potential bias from applying parametric tests to non-normally distributed data (**Table 2**).

**Table 2:** Results of Shapiro-Wilk test for normality of gene expression data.

Gene name	Degrees of freedom (df)	Shapiro-Wilk statistic	P-value
*AKT1*	329	0.96573	5.1e-07
*ALKBH5*	329	0.9914	0.0517
*ELAVL1*	329	0.99305	0.1300
*HNRNPC*	329	0.9853	0.0019

**Figure 6:**
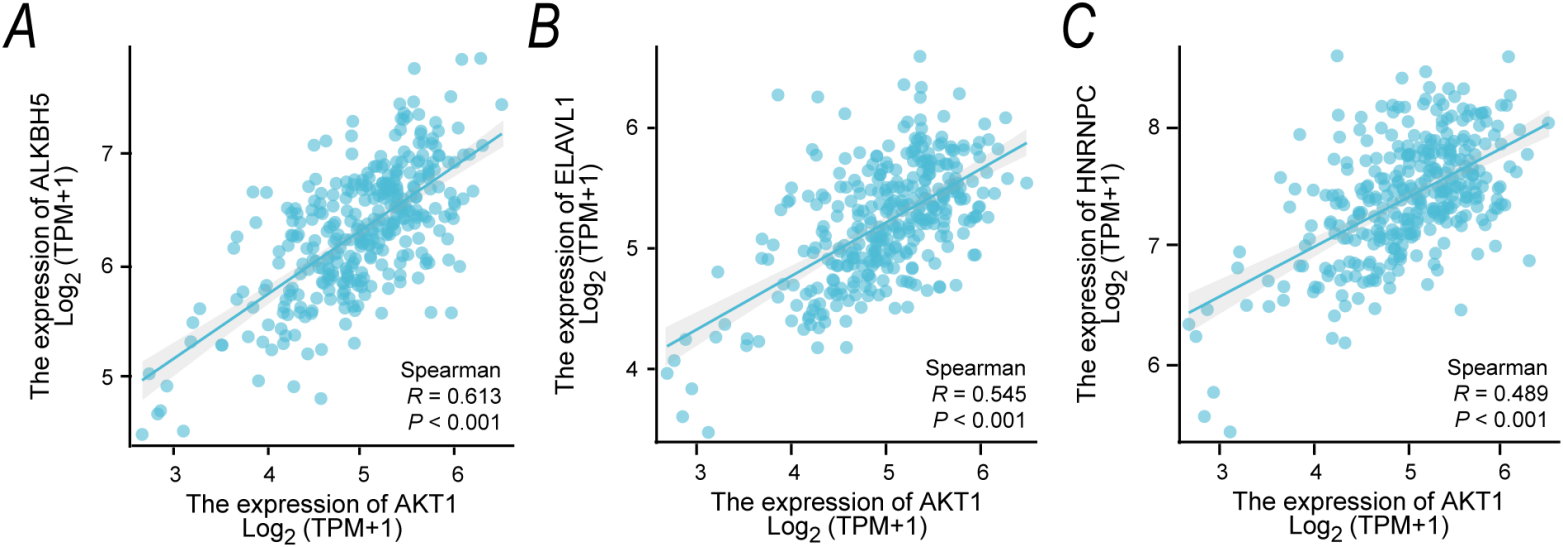
Correlation of AKT1 Expression with m6A Differential Genes in OSCC **(A-C)** Scatter plots showing the correlation between AKT1 and m6A-related differential genes (ALKBH5, ELAVL1, HNRNPC) based on TCGA database OSCC transcriptome data. **(A)** AKT1 vs. ALKBH5, **(B)** AKT1 vs. ELAVL1, **(C)** AKT1 vs. HNRNPC. Spearman correlation analysis was performed, with each point representing a single OSCC sample, and the blue line indicating the linear fitting trend.

The Spearman correlation analysis revealed significant positive correlations between AKT1 and the expression of m6A-regulatory genes ALKBH5, ELAVL1, and HNRNPC. Specifically, the Spearman correlation coefficient between AKT1 and ALKBH5 was R = 0.613 (P < 0.001, **Figure 6A**), between AKT1 and ELAVL1 was R = 0.545 (P < 0.001, **Figure 6B**), and between AKT1 and HNRNPC was R = 0.489 (P < 0.001, **Figure 6C**).

### 3.7 Molecular docking results

As the active ingredient of betel nut, arecoline is rapidly metabolized in oral mucosal cells by esterases to form arecaidine, which has been identified as a key metabolite responsible for the carcinogenic effects of betel nut exposure. To clarify the binding characteristics of arecoline, 4-NQO, and arecaidine with AKT1 during the induction of OSCC, we performed molecular docking studies. The docking results showed binding energies of -5.3 kcal/mol for arecoline, -5.4 kcal/mol for 4-NQO, and -5.1 kcal/mol for arecaidine with AKT1 (**Table 3**).

**Table 3:** Molecular docking binding energies of compounds with ATK1 protein and corresponding database information.

Chemical name	PubChem CID	Uniprot ID	Binding energies (kcal/mol)
arecoline	2230	ATK1 (1UNQ)	-5.3
4-NQO	5955		-5.4
arecaidine	10355		-5.1

**Figure 7:**
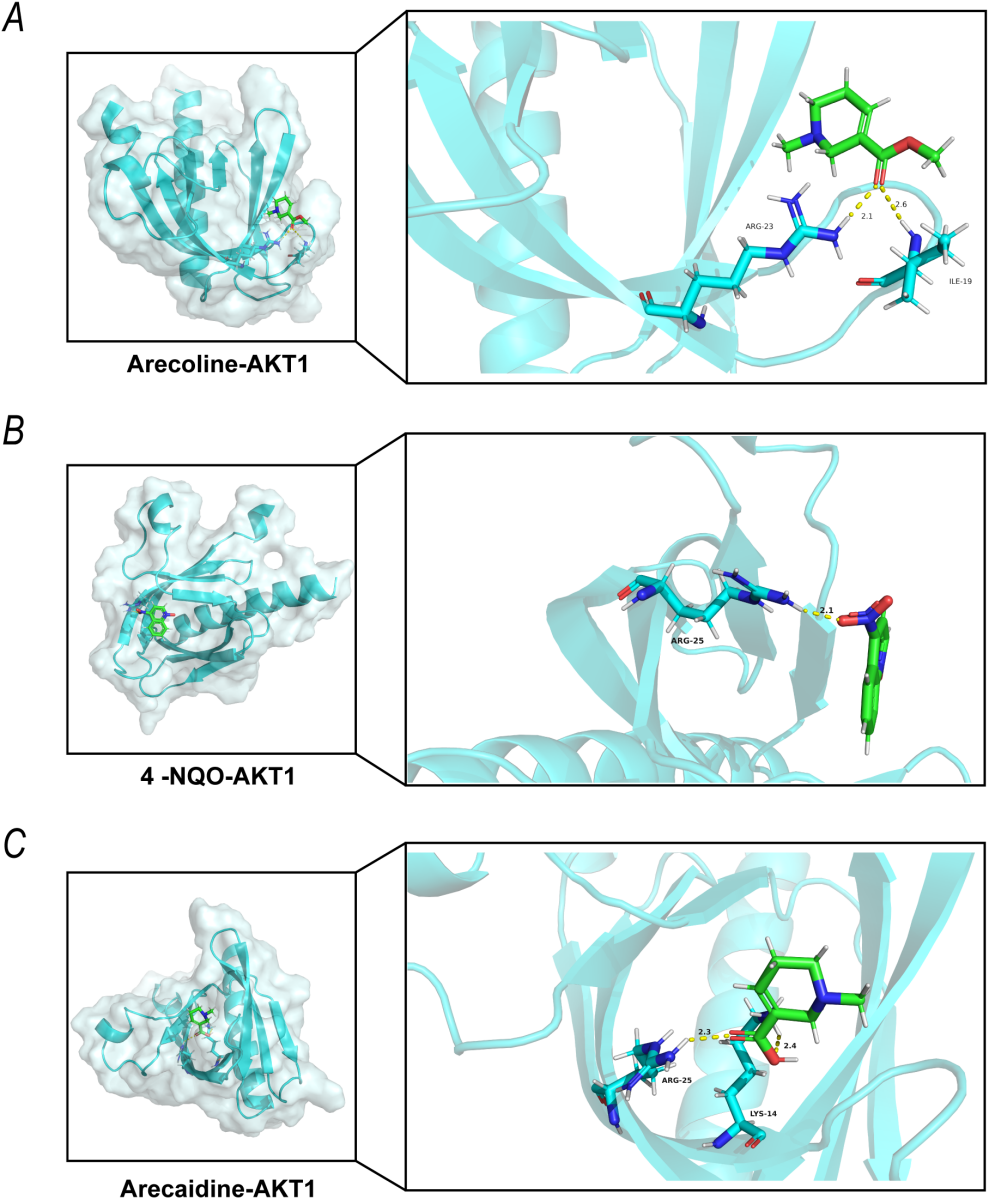
Molecular Docking of Arecoline, 4-NQO, and Arecaidine with AKT1 Schematic Figure. **(A)** The 3D molecular docking conformation and interaction details of arecoline with AKT1 protein; **(B)** The binding mode and key interaction sites of 4-NQO with AKT1 protein; **(C)** The docking results and binding interface analysis of arcadine with AKT1 protein.

Specifically, **Figure 7A** illustrates the binding mode of arecoline with AKT1: arecoline forms a hydrogen bond (2.1 Å) with the ARG-23 residue of AKT1 and interacts with ILE-19 through a hydrogen bond (2.6 Å), stabilizing the binding conformation. **Figure 7B** presents the binding pattern of 4-NQO with AKT1: 4-NQO forms a hydrogen bond (2.1 Å) with ARG-25 of AKT1. **Figure 7C** depicts the binding of arecaidine to AKT1: arecaidine forms a hydrogen bond (2.3 Å) with ARG-25 and interacts with LYS-14 via a hydrogen bond (2.4 Å), supporting its effective binding to AKT1.

## 4 Discussion

OSCC, the predominant malignancy of the head and neck region, is strongly associated with the combined effects of betel nut chewing and tobacco exposure. However, the molecular mechanisms underlying this synergistic carcinogenesis, particularly those related to post-transcriptional regulation such as m6A modifications, remain insufficiently explored. Therefore, this study integrated transcriptomics, network toxicology, and molecular docking approaches to systematically investigate the potential molecular mechanisms involved in OSCC induction by arecoline in combination with 4-NQO. Our study identified HNRNPC, ALKBH5, and ELAVL1 as potential core m6A regulators, revealing that arecoline and 4-NQO can co-modulate OSCC through 494 potential targets. Further analysis showed a significant positive correlation between the core target AKT1 and HNRNPC, ALKBH5, and ELAVL1, with molecular docking confirming that arecoline, its metabolite arecaidine, and 4-NQO can all bind to AKT1 (Schematic figure).

The study found that HNRNPC, ALKBH5, and ELAVL1 are significantly overexpressed in OSCC tissues, with high HNRNPC expression strongly correlated with poor patient prognosis. As an m6A "reader" protein, HNRNPC regulates RNA splicing, transport, and stability by recognizing m6A modification sites on target mRNAs (28–30). Previous studies have shown that HNRNPC is abnormally upregulated in various cancers, promoting tumor progression through multiple mechanisms, such as stabilizing mRNA of oncogenes (e.g., IRAK1), maintaining stability of monocarboxylate transporter MCT1 mRNA to enhance ferroptosis resistance, or modulating immune checkpoint genes such as PD-L1 (31–34). Moreover, in the context of OSCC, the dysregulation of HNRNPC has been linked to clinical diagnosis and prognosis (35, 36). Survival analysis in this study (Kaplan-Meier curve) further confirmed that high HNRNPC expression is associated with poor prognosis in OSCC patients. HNRNPC promotes OSCC cell proliferation, migration, and epithelial-mesenchymal transition (EMT) (37), suggesting that under the stimulation of arecoline and 4-NQO, it may exacerbate m6A-mediated RNA metabolic dysregulation and contribute to the joint carcinogenic process. ALKBH5, an m6A "eraser," removes m6A modifications from mRNA to enhance its stability and regulates tumor progression in various cancers (38, 39). Previous research has linked ALKBH5 to OSCC prognosis, malignant progression, and cisplatin resistance (40–42), and this study further highlights its potential role in arecoline and 4-NQO-induced OSCC. ELAVL1, which stabilizes mRNA by binding to the 3'UTR region, may synergistically cooperate with the inflammatory microenvironment induced by arecoline to maintain the high expression of pro-cancer genes such as VEGF. Taken together, the dysregulation of HNRNPC, ALKBH5, and ELAVL1 is a key epitranscriptomic event in the carcinogenic process associated with concurrent exposure to arecoline and 4-NQO.

In this study, we identified 494 potential targets of arecoline and 4-NQO, which were significantly enriched in processes such as "oxidative stress response" and the "AGE-RAGE signaling pathway," providing molecular insights into their joint carcinogenic effects. Arecoline induce the production of reactive oxygen species (ROS) and activate inflammatory pathways such as NF-κB (43). 4-NQO directly damages DNA, triggering oxidative stress and evasion of apoptosis (44, 45). The enrichment of the "oxidative stress response" pathway suggests that these compounds may concurrently induce ROS, disrupting cellular redox homeostasis and promoting OSCC progression. Furthermore, the enrichment of classic tumor pathways such as PI3K-AKT-mTOR provides clues to the mechanisms through which these compounds regulate cell proliferation and survival. Shared targets may amplify carcinogenic effects through cross-talk between multiple pathways (e.g., oxidative stress and PI3K-AKT pathways), further elucidating the molecular basis of "Areca catechu L.-tobacco co-exposure.

AKT1, a central node in the protein-protein interaction (PPI) network for potential OSCC-inducing targets, is significantly positively correlated with HNRNPC, ALKBH5, and ELAVL1, suggesting that it serves as a key hub mediating "compound-m6A modification-OSCC" interactions. The PI3K-AKT pathway, a core regulatory pathway for cell proliferation and survival, is aberrantly activated in OSCC (46). From a regulatory perspective, arecoline and 4-NQO may bind to AKT1 and activate the PI3K-AKT pathway, directly promoting cellular malignant transformation. On the other hand, we propose a testable hypothesis that AKT1 may indirectly regulate global m6A modification levels by transcriptionally modulating m6A enzymes, thereby influencing the expression of downstream target genes. This "cross-regulation between signaling pathways and m6A modification" could serve as a core molecular mechanism for the co-carcinogenicity of these compounds and provide a theoretical basis for developing combination therapies targeting both AKT1 and m6A modifications. Notably, a study on colorectal cancer has provided direct mechanistic evidence for this regulatory pattern, showing that AKT1 upregulates the expression of the transcription factor RXRA, which, in turn, directly activates the transcription of the m6A demethylase FTO, thereby reducing global RNA m6A levels and inhibiting cancer cell proliferation (47). Although the direct regulatory relationship between the m6A regulators (HNRNPC, ALKBH5, ELAVL1) identified in this study and AKT1 remains to be experimentally verified, the cross-regulation model of "signaling pathways and m6A modifications" that we propose could serve as the core molecular mechanism for the carcinogenesis of arecoline and 4-NQO. This hypothesis will be tested in future functional experiments.

Molecular docking results revealed that 4-NQO, arecoline, and arecaidine bind to AKT1 with binding energies of -5.4 kcal/mol, -5.3 kcal/mol, and -5.1 kcal/mol, respectively, all meeting the effective binding threshold of ≤-5 kcal/mol (in molecular docking, more negative binding energies indicate stronger *in vitro* binding stability between the small molecules and the target protein) (48). Among them, 4-NQO exhibited the best *in vitro* binding stability with AKT1, followed by arecoline. Arecoline, as the core active ingredient in areca nut, are rapidly metabolized into arecoline by esterases in oral mucosal cells. Moreover, the local accumulation of arecaidine in oral mucosa is much higher than that of arecoline. Importantly, previous studies have confirmed that arecaidine has stronger cytotoxicity, promotes oral mucosal fibrosis, and induces EMT more significantly than arecoline (49). Therefore, arecoline, in conjunction with arecaidine, may bind to AKT1 and more efficiently activate the PI3K-AKT pathway and the downstream m6A regulatory network, serving as a key effector molecule in betel nut-induced OSCC.

From an intervention perspective, these findings provide precise guidance for OSCC stratified prevention and treatment. First, for OSCC related to Areca nut chewing, targeting the interaction between arecoline and AKT1 is crucial. Despite its slightly weaker *in vitro* binding stability, as the primary metabolite of arecoline *in vivo*, the development of small-molecule inhibitors that specifically block this interaction could mitigate the carcinogenic effects of arecoline, particularly for primary prevention in regions with high betel nut consumption. Second, the binding conformation between 4-NQO and AKT1 could provide insights for interventions targeting tobacco-related OSCC. Tobacco smoke contains carcinogens similar in structure to 4-NQO, and inhibitors targeting the 4-NQO-AKT1 interaction may offer potential therapeutic targets for OSCC in tobacco-exposed populations.

In conclusion, this study integrated bioinformatics, network toxicology, and molecular docking techniques to construct a multi-dimensional "compound-target-m6A-OSCC" regulatory network, providing novel insights into the molecular mechanisms underlying arecoline/4-NQO-induced OSCC. However, there are still limitations that need to be addressed. Firstly, the proposed regulatory axis (arecoline/4-NQO–AKT1–m6A factors–OSCC) is based on bioinformatics predictions and lacks functional validation through wet-lab experiments. To verify this hypothesis, future studies will employ OSCC cell lines (e.g., CAL-27, SCC-9) to perform AKT1 knockdown (via siRNA or shRNA) and overexpression (via recombinant plasmid transfection) experiments. The primary aims are to: (1) confirm whether AKT1 regulates the mRNA and protein expression levels of key m6A modification factors (HNRNPC, ALKBH5, ELAVL1) as predicted; (2) evaluate the impact of AKT1 modulation on OSCC cell malignant phenotypes (proliferation, migration, invasion, apoptosis) through CCK-8, Transwell, wound-healing, and flow cytometry assays, thereby validating the functional relevance of AKT1 in OSCC progression. Secondly, this study only identifies shared targets and potential joint effects of arecoline and 4-NQO based on in silico analysis, without experimental evidence from combined exposure models. Therefore, no conclusions can be drawn regarding their actual synergistic carcinogenic effects *in vitro* or *in vivo*. Future studies will use combinatorial exposure protocols to directly examine whether arecoline and 4-NQO act synergistically during OSCC development. Thirdly, direct evidence for m6A methylation regulation is lacking, as m6A methylation sequencing (MeRIP-seq) was not performed in this study. Future work will conduct MeRIP-seq to: (1) identify global changes in m6A modification patterns in OSCC cells treated with arecoline and 4-NQO; (2) clarify whether AKT1 mediates m6A modification of specific downstream target mRNAs (as predicted by bioinformatics analysis); (3) verify the regulatory role of m6A enzymes (HNRNPC, ALKBH5, ELAVL1) in targeting these mRNAs, including their effects on mRNA stability and translation efficiency. Collectively, future studies will systematically validate the proposed regulatory mechanisms through the aforementioned wet-lab experiments and expand m6A modification transcriptomics analyses, aiming to more comprehensively and rigorously elucidate the molecular mechanisms of OSCC induced by arecoline and 4-NQO.

## Data Availability

The original contributions presented in the study are included in the article/supplementary material. Further inquiries can be directed to the corresponding author.

## References

[1] Tan Y Wang Z Xu M Li B Huang Z Qin S . Oral squamous cell carcinomas: state of the field and emerging directions. Int J Oral Sci. (2023) 15:44. doi: 10.1038/s41368-023-00249-w, PMID: 37736748 PMC10517027

[2] Bray F Laversanne M Sung H Ferlay J Siegel RL Soerjomataram I . Global cancer statistics 2022: GLOBOCAN estimates of incidence and mortality worldwide for 36 cancers in 185 countries. CA: Cancer J Clin. (2024) 74:229–63. doi: 10.3322/caac.21834, PMID: 38572751

[3] Rangani SC Marapana RAUJ Senanayake GSA Perera PRD Pathmalal MM Amarasinghe HK . Alkaloids and nitrosamines in betel quid: A biochemical exploration of carcinogenicity. Chem-Biol Interact. (2025) 407:111383. doi: 10.1016/j.cbi.2025.111383, PMID: 39805416

[4] Marques MM Beland FA Lachenmeier DW Phillips DH Chung FL Dorman DC . Carcinogenicity of acrolein, crotonaldehyde, and arecoline. Lancet Oncol. (2021) 22:19–20. doi: 10.1016/S1470-2045(20)30727-0, PMID: 33248467

[5] Kowalski LP Oliveira MM Lopez RVM Silva D Ikeda MK Curado MP . Survival trends of patients with oral and oropharyngeal cancer treated at a cancer center in S o Paulo, Brazil. Clinics. (2020) 75:e1507. doi: 10.6061/clinics/2020/e1507, PMID: 32294669 PMC7134554

[6] Sahu SR Thakur S Peroumal D Utkalaja BG Dutta A Kumari P . 4-nitroquinoline 1-oxide induces immune cells death to onset early immunosuppression during oral squamous cell carcinoma development. Front Immunol. (2023) 14:1274519. doi: 10.3389/fimmu.2023.1274519, PMID: 37936711 PMC10626482

[7] Spuldaro TR Wagner VP N r F Gaio EJ Squarize CH Carrard VC . Periodontal disease affects oral cancer progression in a surrogate animal model for tobacco exposure. Int J Oncol. (2022) 60. doi: 10.3892/ijo.2022.5367, PMID: 35514311 PMC9097773

[8] Zhang P Zhao WL Li JK Tong JY . Sichuan da xue xue bao. Yi xue ban = Journal of Sichuan University. Medical Science Edition. (2022) 53:1118–26. doi: 10.12182/20221160511, PMID: 36443062 PMC10408978

[9] Liu Y Yang D Liu T Chen J Yu J Yi P . N6-methyladenosine-mediated gene regulation and therapeutic implications. Trends Mol Med. (2023) 29:454–67. doi: 10.1016/j.molmed.2023.03.005, PMID: 37068987

[10] Jayaprakash JP Karemore P Khandelia P . METTL3 promotes oral squamous cell carcinoma by regulating miR-146a-5p/SMAD4 axis. Oncotarget. (2025) 16:291–309. doi: 10.18632/oncotarget.28717, PMID: 40338154 PMC12060920

[11] Wang Z Li H Cai H Liang J Jiang Y Song F . FTO Sensitizes Oral Squamous Cell Carcinoma to Ferroptosis via Suppressing ACSL3 and GPX4. Int J Mol Sci. (2023) 24. doi: 10.3390/ijms242216339, PMID: 38003537 PMC10671523

[12] Zhang J Li H Zhang Y Huang J Ren L Zhang C . Computational toxicology in drug discovery: applications of artificial intelligence in ADMET and toxicity prediction. Brief Bioinform. (2025) 26. doi: 10.1093/bib/bbaf533, PMID: 41052279 PMC12499773

[13] Zheng G Lu M Hu B Chen S Xu S Sun G . Emerging plasticizer induced lipid metabolism disorders revealed by network toxicology molecular docking and dynamics simulation. Scientific Reports. (2025) 15:34559. doi: 10.1038/s41598-025-17931-0, PMID: 41044206 PMC12494788

[14] Cheng W Lin P Yang Z Xie Y Gao D Chen M . A new perspective on the neurotoxic mechanisms of six typical per- and polyfluoroalkyl substances (PFAS): insights from integrating network toxicology and random forest algorithm. Drug Chem Toxicol. (2026) 49:130–48. doi: 10.1080/01480545.2025.2572631, PMID: 41088807

[15] Yu G Li F Qin Y Bo X Wu Y Wang S . GOSemSim: an R package for measuring semantic similarity among GO terms and gene products. Bioinformatics. (2010) 26:976–8. doi: 10.1093/bioinformatics/btq064, PMID: 20179076

[16] Rong Y Liu X Sun Y Yang L Chen L . Identification of empagliflozin-related hub genes in atherosclerosis and their correlations with immune infiltration: Network pharmacology and bioinformatics analyses. PLOS One. (2026) 21:e0339956. doi: 10.1371/journal.pone.0339956, PMID: 41544081 PMC12810795

[17] Liu J Li X Lu X Zhang Z Li C . Identification of neurotrophic factor related biomarkers and mechanistic insights into neuropathic pain via integrated bioinformatics analysis. ACS Omega. (2025) 10:57418–31. doi: 10.1021/acsomega.5c07765, PMID: 41358093 PMC12676309

[18] Keiser MJ Roth BL Armbruster BN Ernsberger P Irwin JJ Shoichet BK . Relating protein pharmacology by ligand chemistry. Nat Biotechnol. (2007) 25:197–206. doi: 10.1038/nbt1284, PMID: 17287757

[19] Gfeller D Michielin O Zoete V . Shaping the interaction landscape of bioactive molecules. Bioinformatics. (2013) 29:3073–9. doi: 10.1093/bioinformatics/btt540, PMID: 24048355

[20] Daina A Zoete V . Testing the predictive power of reverse screening to infer drug targets, with the help of machine learning. Comm Chem. (2024) 7:105. doi: 10.1038/s42004-024-01179-2, PMID: 38724725 PMC11082207

[21] Daina A Michielin O Zoete V . SwissTargetPrediction: updated data and new features for efficient prediction of protein targets of small molecules. Nucleic Acids Res. (2019) 47:W357–64. doi: 10.1093/nar/gkz382, PMID: 31106366 PMC6602486

[22] Yao ZJ Dong J Che YJ Zhu MF Wen M Wang NN . TargetNet: a web service for predicting potential drug-target interaction profiling via multi-target SAR models. J Comp-Aided Mol Des. (2016) 30:413–24. doi: 10.1007/s10822-016-9915-2, PMID: 27167132

[23] Wiegers TC Davis AP Wiegers J Sciaky D Barkalow F Wyatt B . Integrating AI-powered text mining from PubTator into the manual curation workflow at the Comparative Toxicogenomics Database. Database. (2025) 2025. doi: 10.1093/database/baaf013, PMID: 39982792 PMC11844237

[24] Zdrazil B Felix E Hunter F Manners EJ Blackshaw J Corbett S . The ChEMBL Database in 2023: a drug discovery platform spanning multiple bioactivity data types and time periods. Nucleic Acids Res. (2024) 52:D1180–92. doi: 10.1093/nar/gkad1004, PMID: 37933841 PMC10767899

[25] Davies M Nowotka M Papadatos G Dedman N Gaulton A Atkinson F . ChEMBL web services: streamlining access to drug discovery data and utilities. Nucleic Acids Res. (2015) 43:W612–20. doi: 10.1093/nar/gkv352, PMID: 25883136 PMC4489243

[26] Wang X Shen Y Wang S Li S Zhang W Liu X . PharmMapper 2017 update: a web server for potential drug target identification with a comprehensive target pharmacophore database. Nucleic Acids Res. (2017) 45:W356–60. doi: 10.1093/nar/gkx374, PMID: 28472422 PMC5793840

[27] Kong X Liu C Zhang Z Cheng M Mei Z Li X . BATMAN-TCM 2.0: an enhanced integrative database for known and predicted interactions between traditional Chinese medicine ingredients and target proteins. Nucleic Acids Res. (2024) 52:D1110–20. doi: 10.1093/nar/gkad926, PMID: 37904598 PMC10767940

[28] Shetty S . Regulation of urokinase receptor mRNA stability by hnRNP C in lung epithelial cells. Mol Cell Biochem. (2005) 272:107–18. doi: 10.1007/s11010-005-7644-2, PMID: 16010978

[29] McCloskey A Taniguchi I Shinmyozu K Ohno M . hnRNP C tetramer measures RNA length to classify RNA polymerase II transcripts for export. Science. (2012) 335:1643–6. doi: 10.1126/science.1218469, PMID: 22461616

[30] Fischl H Neve J Wang Z Patel R Louey A Tian B . hnRNPC regulates cancer-specific alternative cleavage and polyadenylation profiles. Nucleic Acids Res. (2019) 47:7580–91. doi: 10.1093/nar/gkz461, PMID: 31147722 PMC6698646

[31] Yang G Shen L Cui M Yang J . Novel RNA-methylase HNRNPC promotes gastric cancer tumorigenesis by triggering the lactate-induced ferroptosis resistance. Front Immunol. (2025) 16:1612935. doi: 10.3389/fimmu.2025.1612935, PMID: 40977716 PMC12443684

[32] Liu YY Xia M Chen ZB Liao YD Zhang CY Yuan L . HNRNPC mediates lymphatic metastasis of cervical cancer through m6A-dependent alternative splicing of FOXM1. Cell Death & Disease. (2024) 15:732. doi: 10.1038/s41419-024-07108-4, PMID: 39375330 PMC11458786

[33] Chen JJ Lu TZ Wang T Yan WH Zhong FY Qu XH . The m6A reader HNRNPC promotes glioma progression by enhancing the stability of IRAK1 mRNA through the MAPK pathway. Cell Death & Disease. (2024) 15:390. doi: 10.1038/s41419-024-06736-0, PMID: 38830885 PMC11148022

[34] Fang C Wu Y Wu Y Guo Q Wu J Ke G . The effectiveness of anti-PD-L1 treatment and the underlying regulatory mechanism in ovarian clear cell carcinoma. International Immunopharmacology. (2025) 164:115344. doi: 10.1016/j.intimp.2025.115344, PMID: 40803218

[35] Lu CH Yin XL Huang ZD Lv SA Wu J Wei J . Bioinformatics identification and validation of m6A/m1A/m5C/m7G/ac4C-modified genes in oral squamous cell carcinoma. BMC Cancer. (2025) 25:1055. doi: 10.1186/s12885-025-14216-7, PMID: 40597017 PMC12211329

[36] Yu M Shen F Li X Zhu C Pei Y Zhang M . Bioinformatics analysis of the prognosis and biological significance of N6 methyladenine regulators in oral squamous cell carcinoma. J Gene Med. (2024) 26:e3619. doi: 10.1002/jgm.3619, PMID: 37985224

[37] Jia R Che X Jia J Guo J . FOXM1a isoform of oncogene FOXM1 is a tumor suppressor suppressed by hnRNP C in oral squamous cell carcinoma. Biomolecules. (2023) 13:1331. doi: 10.3390/biom13091331, PMID: 37759731 PMC10526205

[38] Huang B Zhang X Chen J Wang H . ALKBH5: a double-edged sword in cancer ferroptosis regulation: A review. Int J Biol Macromol. (2025) 330:147999. doi: 10.1016/j.ijbiomac.2025.147999, PMID: 41033518

[39] Zhang X Zhu C Huang B Wang H . The dual-edged sword: AlkB homolog 5-mediated autophagy regulation in cancers - molecular mechanisms and therapeutic implications.Int J Biol Macromol. (2025) 321:146227. doi: 10.1016/j.ijbiomac.2025.146227, PMID: 40701469

[40] Shriwas O Priyadarshini M Samal SK Rath R Panda S Das Majumdar SK . DDX3 modulates cisplatin resistance in OSCC through ALKBH5-mediated m6A-demethylation of FOXM1 and NANOG. Apoptosis. (2020) 25:233–46. doi: 10.1007/s10495-020-01591-8, PMID: 31974865

[41] Liu Y Nie J Huang Y Yang Y Su W Zhang Y . m6A-related genes ALKBH5 and RBMX as prognostic and progression biomarkers in Chinese oral squamous cell carcinoma patients. Arch Oral Biol. (2025) 170:106149. doi: 10.1016/j.archoralbio.2024.106149, PMID: 39643954

[42] Udompatanakorn C Sriviriyakul P Taebunpakul P . A study of RNA m6A demethylases in oral epithelial dysplasia and oral squamous cell carcinoma. J Oral Biol Craniofacial Res. (2023) 13:111–6. doi: 10.1016/j.jobcr.2022.12.003, PMID: 36582218 PMC9792536

[43] Wang TH Shen YW Chen HY Chen CC Lin NC Shih YH . Arecoline induces ROS accumulation, transcription of proinflammatory factors, and expression of KRT6 in oral epithelial cells. Biomedicines. (2024) 12. doi: 10.3390/biomedicines12020412, PMID: 38398015 PMC10887121

[44] Ornstein RL Rein R . Molecular models of induced DNA premutational damage and mutational pathways for the carcinogen 4-nitroquinoline 1-oxide and its metabolites. Chem-Biol Interact. (1980) 30:87–103. doi: 10.1016/0009-2797(80)90117-9, PMID: 6769596

[45] Han H Pan Q Zhang B Li J Deng X Lian Z . 4-NQO induces apoptosis via p53-dependent mitochondrial signaling pathway. Toxicology. (2007) 230:151–63. doi: 10.1016/j.tox.2006.11.045, PMID: 17169477

[46] Veerasamy V Veeran V Nagini S . Dysregulated PI3K/AKT signaling in oral squamous cell carcinoma: the tumor microenvironment and epigenetic modifiers as key drivers. Oncol Res. (2025) 33:1835–60. doi: 10.32604/or.2025.064010, PMID: 40746882 PMC12308251

[47] Ren Q Xiang M Qiao J Liu Z Zhang G Gu L . TTC7B triggers the PI4KA-AKT1-RXRA-FTO axis and inhibits colon cancer cell proliferation by increasing RNA methylation. Int J Biol Sci. (2025) 21:1127–43. doi: 10.7150/ijbs.102431, PMID: 39897037 PMC11781174

[48] Pantsar T Poso A . Binding affinity via docking: fact and fiction. Molecules. (2018) 23:1899. doi: 10.3390/molecules23081899, PMID: 30061498 PMC6222344

[49] Warnakulasuriya S Chen THH . Areca nut and oral cancer: evidence from studies conducted in humans. J Dental Res. (2022) 101:1139–46. doi: 10.1177/00220345221092751, PMID: 35459408 PMC9397398

